# Effects of Time-Dependent Protein Restriction on Growth Performance, Digestibility, and mTOR Signaling Pathways in Juvenile White Shrimp *Litopenaeus vannamei*

**DOI:** 10.3389/fphys.2021.661107

**Published:** 2021-04-09

**Authors:** Wei Zhao, Hongjie Luo, Wanqing Zhu, Xiaoqin Yuan, Jianchun Shao

**Affiliations:** ^1^Key Laboratory of Marine Biotechnology of Fujian Province, Institute of Oceanology, Fujian Agriculture and Forestry University, Fuzhou, China; ^2^Department of Ecology, Institute of Hydrobiology, College of Life Science and Technology, Jinan University, Guangzhou, China; ^3^Laboratory for Marine Biology and Biotechnology, Qingdao National Laboratory for Marine Science and Technology, Qingdao, China

**Keywords:** protein restriction, feeding strategy, growth performance, digestibility, mTOR signaling pathway, *Litopenaeus vannamei*

## Abstract

A 6-week feeding strategy experiment was conducted to investigate the effects of time-dependent protein restriction and subsequent recovery on shrimp. Diets with protein levels of 43 and 36% were used as adequate and restricted diets, respectively. Shrimp with an initial body weight of 6.52 ± 0.46 g were given four feeding strategies: feeding on an adequate diet for six weeks (T1, the control), having protein-restricted diet in weeks 1 and 4 (T2), being given a protein-restricted diet in weeks 1, 3, and 5 (T3), and having protein-restricted diet in weeks 1, 2, 4, and 5 (T4). WG, SGR, FE, and PER of shrimp in T1–T3 showed no significant difference (*P* > 0.05), these indicators of T4 were significantly reduced (*P* < 0.05). No significant differences were found in digestive enzyme activities of shrimp among all treatments (*P* > 0.05). Crude protein content of shrimp muscle in T4 was lower than that of T1–T3. The expression level of *tor* in T4 was lower than that in other treatments, while *4e-bp* was higher than that of other treatments. To balance saving on feeding cost and growth performance, giving the shrimp a protein-restricted diet for 1 week with subsequent refeeding (T2 and T3) is suitable for shrimp under high-density conditions.

## Introduction

The success of culturing shrimp depends on maximizing the cost-effectiveness of production. Feed is considered as the most important item in the total production cost of aquaculture (Yokoyama et al., 2009). Protein is the most important and expensive ingredient in formulated feed, and dietary protein level is the primary factor influencing shrimp growth and feed costs (Aghaei et al., 2018; Xie et al., 2020b). Enhanced growth performance resulting from optimal dietary protein level has been reported in fish, such as *Ctenopharyngodon idella* (Xu et al., 2016), *Solea senegalensis* (Rema et al., 2008) and *Cyprinus carpio* (Liu et al., 2009), and crustaceans, such as *Litopenaeus vannamei* (Xia et al., 2010), *Scylla serrata* (Catacutan, 2002), and *Portunus trituberculatus* (Jin et al., 2013). However, if excess protein is provided, it will be metabolized as an energy source rather than used for growth, and excess protein may increase the excretion of nitrogen into the aquatic environment, thus reducing water quality and growth performance (Ali et al., 2003; Dong et al., 2013). Therefore, an appropriate protein management strategy is particularly important for the sustainable development of aquaculture industry. At present, it is very popular to use plant protein instead of fish meal or to increase the level of lipids or carbohydrates in the diet to spare dietary protein (Ding et al., 2015; García-Ortega et al., 2016; Xie et al., 2016; Salas-Leiton et al., 2018; Mock et al., 2019). Compared with these dietary protein management strategies, the use of protein-restricted feeding to improve protein utilization has also attracted the attention of aquatic nutritionists.

An animal’s ability to grow extremely rapidly after the growth inhibition stage due to feed restriction and adverse environmental conditions is referred to as compensatory growth (Jobling, 2010; Won and Borski, 2013). The response of compensatory growth after short-term feed restriction is mainly due to the improvement of feed efficiency by better digestion and absorption of nutrients, and metabolism and energy consumption caused by reduced exercise (Ali et al., 2003). The research on the compensatory growth of aquatic animals has become a prominent subject because it not only helps to save on feed costs, but it also helps to alleviate the pressure of water environment caused by excessive use of feed in aquaculture process (Zhu et al., 2016; Mohanta et al., 2017; Stumpf et al., 2019). Strategies for short-term restriction or deprivation of feed have been adopted by aquaculturists to reduce mortality from disease outbreaks or to solve water quality problems (Caruso et al., 2011).

To date, two major restrictive feeding strategies were widely used, including short-term fasting, refeeding, and protein-restricted feeding. Short-term fasting and refeeding can produce the compensatory growth phenomenon that has been reported in *Lophiosilurus alexandri* (Silva et al., 2019), *Barbonymus schwanenfeldii* (Eslamloo et al., 2012), *Acipenser baerii* (Morshedi et al., 2013), *Fenneropenaeus chinensis* (Zhang et al., 2010), *Cherax quadricarinatus* (Stumpf et al., 2011, 2014), and *Penaeus vannamei* (Shao et al., 2020). However, the unsatisfactory performance of short-term fasting and refeeding has also been reported widely: fish subjected to short-term fasting and refeeding were unable to maintain the same growth rate as control fish (Eroldoğan et al., 2008; Peres et al., 2011; Takahashi et al., 2011). However, fasting may cause a decline in growth and immune function and increase the risk of disease outbreaks (Caruso et al., 2011; Khalil et al., 2016). Thus, protein restriction with subsequent refeeding may be a more effective feeding strategy than fasting and refeeding, because it does not make a significant inhibitory effect on the physiological function of fish or shrimp. Protein restriction with subsequent refeeding has been reported to produce compensatory growth in fish and shrimp (Wu and Dong, 2002; Sevgili et al., 2012; Dong et al., 2013; Aghaei et al., 2018). Therefore, a reasonable protein restriction feeding strategy can effectively improve the income of aquaculturists and alleviate the environmental pollution caused by aquaculture activities.

The white shrimp *L. vannamei* is the most extensively cultured crustacean species in the world. Culture of *L. vannamei* has also become an economically important aquaculture activity in China due to its high nutritional value, yield and market demand (Zhao et al., 2018; Xie et al., 2020a). Therefore, an appropriate protein restriction feeding strategy is particularly important for saving on feed costs and increasing the income of shrimp farmers. However, studies on the effects of time-dependent protein restriction on *L. vannamei* have not been reported. The optimum protein level of *L. vannamei* in high-density cultures is around 43%, which correlates with optimal growth performance (Xia et al., 2010). At present, the protein level of commercial formula feed for *L. vannamei* produced in China is around 43%. Considering the protein level of commercial *L. vannamei* formula feed, a diet with a 43% protein level is used as an adequate diet, and diet of 36% protein is used as protein-restricted diet. These two protein levels were used to assess the effects of time-dependent protein restriction on *L. vannamei*. Therefore, this study was designed to determine the effects of time-dependent protein restriction on growth performance, digestibility, and growth-related mTOR signaling pathway. The data obtained in this study can provide reference for feeding strategies in the shrimp culture process.

## Materials and Methods

### Shrimp and Diet Preparation

Shrimp were purchased from an aquatic farm in Qingdao City, China. Prior to the experiment, the shrimp were acclimatized for a week. The water temperature was maintained at 28 ± 1°C, and the salinity was maintained at 30 ppt.

Two isolipidic (80 g kg^–1^) diets containing 36.0% (protein-restricted diet) and 43% (protein-adequate diet) crude protein (dry weight basis) were prepared, and proximate composition analysis of the diets was presented in Table 1. Fish meal and soybean meal were used as the main protein sources, and fish oil and soybean lecithin were used as the main lipid sources. All dry ingredients were finely ground to obtain a particle size <300 μm and thoroughly mixed and homogenized. The 1.6-mm diameter pellets were produced by using a meat grinder. The pellets were then dried in a forced air oven at 40°C for 24 h to approximately 10% moisture content and stored at −20°C until used.

**TABLE 1:** Composition of diets formulated to contain 36 and 43% crude protein.

Ingredient (g/100g)	43%	36%
Fish meal^a^	40	30
Soybean meal^b^	30	22.5
Wheat flour^c^	21.9	38.4
Fish oil	2.6	3.6
Soybean lecithin	1	1
Brewer’s yeast	2.5	2.5
Vitamin premix^d^	1	1
Mineral premix^e^	1	1
**Proximate analysis**		
Moisture	9.36	9.12
Crude protein	43.28	36.12
Crude lipid	7.86	8.04
Ash	11.46	12.13
Gross energy (kJ g^−1^)	16.91	16.31

*^*a*^Fish meal: 64.1% crude protein, 9.5% crude lipid, Dale Feed Corporation, Yantai, China. ^*b*^Soybean meal: 42.8% crude protein, 1.2% crude lipid, Dale Feed Corporation, Yantai, China. ^*c*^Wheat flour: 13.0% crude protein, 0.8% crude lipid, Dale Feed Corporation, Yantai, China. ^*d*^Vitamin premix (kg^–1^ of diet): vitamin A, 250,000 IU; riboflavin, 750 mg; pyridoxine-HCl, 400 mg; cyanocobalamin, 1 mg; thiamin, 250 mg; menadione, 250 mg; folic acid, 125 mg; biotin, 10 mg; a-tocopherol, 2.5 g; myo-inositol, 8,000 mg; calcium pantothenate, 1,250 mg; nicotinic acid, 2,000 mg; choline chloride, 8,000 mg; vitamin D3, 45,000 IU; vitamin C, 7,000 mg. ^*e*^Mineral premix (kg^–1^ of diet): ZnSO_4_⋅7H_2_O, 0.04 g; CaCO_3_, 37.9 g; KCl, 5.3 g; KI, 0.04 g; NaCl, 2.6 g; CuSO_4_⋅5H_2_O, 0.02 g; CoSO_4_⋅7H_2_O, 0.02 g; FeSO_4_⋅7H_2_O, 0.9 g; MnSO_4_⋅H_2_O, 0.03 g; MgSO_4_⋅7H_2_O, 3.5 g; Ca (HPO_4_)_2_⋅2H_2_O, 9.8 g.*

### Experimental Procedure

After acclimation, the shrimp were made to fast for 24 h and weighed. Then, 960 shrimp with an initial body weight of 6.52 ± 0.46 g were randomly assigned into 12 fiberglass tanks (water volume: 400 L, 200 shrimp m^–3^). The design was completely randomized with three replicates of each of four feeding treatments: a feeding of 43% protein in the diet for 6 weeks (T1, the control); a feeding with a 36% protein level in weeks 1 and 4 and a feeding with a 43% protein level in the diet in weeks 2, 3, 5, and 6 (T2); a feeding of 36% protein in the diet in weeks 1, 3, and 5, along with a feeding level of 43% protein in weeks 2, 4, and 6 (T3); and a feeding of protein at a level of 36% in the diet in weeks 1, 2, 4, and 5, along with a feeding level of 43% protein in weeks 3 and 6 (T4) (Table 2). During the experiment period, the shrimp were fed four times a day, at 7:00, 12:00, 18:00, and 23:00, at 5% biomass per day. During the period of the experiment, water temperature was maintained at 28 ± 1°C and pH between 7.8 and 8.2, while salinity ranged from 29 ppt to 31 ppt, dissolved oxygen above 6.0 mg L^–1^, ammonia-nitrogen below 0.30 mg L^–1^, and nitrite-nitrogen below 0.10 mg L^–1^. A half of the seawater was replaced daily to remove uneaten diet and feces. Uneaten diet particles were dried at 70°C and weighed and used to calculate feed intake. Every morning before feeding, we sorted out the dead shrimp, and shrimp death numbers were recorded to calculate the survival rate.

**TABLE 2:** Distribution of feed protein levels at different experimental times.

	Feed Protein Levels	
Treatments	36%	43%
T1	–	6 weeks
T2	Weeks 1 and 4	Week 2, 3, 5, and 6
T3	Weeks 1, 3, and 5	Week 2, 4, and 6
T4	Weeks 1, 2, 4, and 5	Weeks 3 and 6

### Sample Collection and Growth Performance Analysis

At the end of the feeding trial, all shrimp were starved for 24 h so as to enter a basic metabolic state and eliminate the dietary effect. All survival shrimps in each tank were collected and anesthetized with MS-222 (Sigma, St Louis, MO, United States), and then weighed and counted to calculate the weight gain (WG), specific growth ratio (SGR), feed efficiency (FE), and protein efficiency ratio (PER). Twelve shrimps from each tank were randomly collected and aseptically sacrificed in ice-bath, and their hepatopancreas and muscle were rapidly frozen in liquid nitrogen and stored at −80°C. Hepatopancreas samples were used to determine digestive enzyme activity (trypsin, α-amylase, and lipase) and digestive enzyme genes (*trypsin* and α*-amylase*) expression level. Muscle samples were used to determine the expression level of genes related to the mTOR signaling pathway. Ten shrimps from each tank were randomly sampled and stored at −20°C for body composition analysis. All the experiments were conducted in accordance with the recommendations in the Guide for the Care and Use of Laboratory Animals of the National Institutes of Health. The study protocol and all experimental procedures were approved by Experimental Animal Ethics Committee of Fujian Agriculture and Forestry University.

WG, SGR, PER, FE, and survival were calculated for each tank according to the following equations:

$$\begin{array}{l} \text{WG}\left(\%\right)=100\times \left({\text{W}}_{2}-W_{1}\right)/{\text{W}}_{1}\\ \mathrm{SGR}\left(\%{\mathrm{day}}^{-1}\right)=100\times \left({\mathrm{lnW}}_{2}-{\text{lnW}}_{1}\right)/\text{t}\\ \mathrm{Survival}\left(\%\right)=100\times {\text{N}}_{\text{f}}/{\text{N}}_{0}\\ \text{FE}=\left(W_{t}-W_{0}\right)/\mathrm{total}\mathrm{dry}\mathrm{feed}\mathrm{intake}\\ \text{PER}=100\times \left(W_{t}-{\text{W}}_{0}\right)/\left(I\times C_{Nf}\right) \end{array}$$

where W_2_ and W_1_ were mean final and initial fish body weights (g); *t* is the duration of the experiment (42 days); N*_*f*_* is number of shrimps at the end of the experiment and N_0_ at the start. W*_*t*_* (g) is total final body weight and W_0_ (g) total initial body weight; C*_*Nf*_* (%) is protein content in the feed; I (g) is the total amount of feed provided on a dry weight basis.

### Chemical Analysis of Feed and Shrimp Muscle

The crude protein, crude lipid, crude ash and moisture of shrimp muscle and experimental diets were analyzed by the Association of Official Analytical Chemists (AOAC, 2012). Moisture was determined by drying in an oven at 105°C for 24 h. Total nitrogen content was determined according to the Kjeldahl method, and crude protein content was calculated in an indirect manner (nitrogen × 6.25). Crude lipids were measured after diethyl ether extraction using the Soxhlet method. Crude ash was examined after combustion in a muffle furnace at 550°C for 24 h. Gross energy content was measured by microbomb calorimeter.

### Activity Quantification of Digestive Enzymes

Hepatopancreas samples were homogenized in ice-cold 50 mM Tris-HCl buffer solution (pH 7.4) in a proportion of 1:9 (w/v) and then centrifuged at 12,000 *g* for 15 min at 4°C; the cold hepatopancreas supernatant was used for evaluation of enzymatic activity. Trypsin activity was measured according to its ability to catalyze the hydrolysis of ester chains of arginine ethyl ester. And α-amylase activity was measured using iodine to reveal non-hydrolyzed starch. Lipase activity was measured by the measurement of fatty acids released by the enzymatic hydrolysis of triglycerides in a stabilized emulsion of olive oil. The activities of trypsin, α-amylase, and lipase were measured using a commercial kit (Nanjing Jiancheng Bioengineering Institute, Nanjing, China).

### Gene Expression Analysis

Total RNA was extracted from the hepatopancreas and muscle using a Reagent kit (Takara, Japan) according to the manufacturer’s instructions. Total RNA concentration and quality were measured by NanoDrop spectrophotometer (ND-2000, Thermo Fisher, United States), and 1.0% agarose gel electrophoresis, respectively. Subsequently, cDNA was synthesized using a TransScript^®^ One-Step gDNA Removal and cDNA Synthesis Kit (TransGen Biotech Co., Ltd., China), according to the manufacturer’s instructions. The amplification was performed by thermal cycler (A300, LongGene, China) in the following stages: 65°C for 5 min, ice bath for 2 min, 42°C for 15 min, and 85°C for 5 s.

RNA extracted from the muscle was used for expression analysis of mTOR signaling pathway genes *tor*, *s6k*, and *4e-bp*, and RNA extracted from the hepatopancreas was used for expression analysis of the digestive enzyme genes *trypsin* and α*-amylase*. The specific primer sequence for real-time quantitative PCR was designed in accordance with Shao et al. (2018) and Sha et al. (2016), as well as β-actin primers for measuring the endogenous control gene (Table 3). Real-time PCR was performed according to the method of Zhao et al. (2017). The dissociation curve analysis was performed at the end of each PCR reaction to confirm that only one PCR product was amplified and measured. The 2^−ΔΔCt^ method was used to analyze the expression level of different genes.

**TABLE 3:** Primers used for real-time quantitative PCR.

Gene name	Primer sequence (5n′–3′)	Product size (bp)
*β-actin*	F-GCCCATCTACGAGGGATA R-GGTGGTCGTGAAGGTGTAA	121
*tor*	F-TGCCAACGGGTGGTAGA R-GGGTGTTTGTGGACGGA	181
*s6k*	F-GCAAGAGGAAGACGCCATA R-CCGCCCTTGCCCAAAACCT	210
*4e-bp*	F-ATGTCTGCTTCGCCCGTCGCTCGCC R-GGTTCTTGGGTGGGCTCTT	226
*trypsin*	F-CGGAGAGCTGCCTTACCAG	141
	R-TCGGGGTTGTTCATGTCCTC	
*α-amylase*	F-CTCTGGTAGTGCTGTTGGCT	116
	R-TGTCTTACGTGGGACTGGAAG	

### Statistical Analysis

All data were presented as mean ± standard deviation. Statistical analyses were conducted using SPSS 19.0 (SPSS, Chicago, IL, United States) and checked for normality and homogeneity of variance before analysis. The growth performance, activity of digestive enzymes, proximate composition of muscle, and gene expression levels were subjected to one-way analysis of variance (ANOVA). The differences among treatment means were resolved using Duncan’s test for unplanned multiple comparisons of the mean. *P* values <0.05 were considered statistically significant.

## Results

### Growth Performance

At the end of the feeding experiment, the growth-related parameters in all experiments were affected by different feeding strategies (*P* < 0.05, Table 4). The final body weights (FBW), GW, SGR, FE, and PER of shrimp in the T1, T2, and T3 treatments were significantly higher than those of shrimp in the T4 treatment (*P* < 0.05). Shrimp in the T2 and T3 treatments showed the highest value of survival rate and significantly higher than that of shrimp in the T1 treatment (*P* < 0.05) but without significant differences from shrimp in the T4 treatment (*P* > 0.05).

**TABLE 4:** Effects of different feeding strategies on growth performance of *Litopenaeus vannamei.*

Items	IBW/g	FBW/g	WG/%	SGR/(%/d)	Survival/%	FI/g	FE	PER
T1	6.52 ± 0.02	14.81 ± 1.14a	127.21 ± 17.45a	1.95 ± 0.19a	82.08 ± 9.04b	12.56 ± 0.82	0.66 ± 0.09a	1.54 ± 0.21a
T2	6.54 ± 0.06	14.14 ± 0.45a	116.85 ± 6.96a	1.84 ± 0.08a	95.00 ± 2.5a	12.21 ± 0.73	0.62 ± 0.04a	1.52 ± 0.09a
T3	6.48 ± 0.04	14.26 ± 0.98a	118.82 ± 15.09a	1.86 ± 0.16a	94.17 ± 4.39a	11.86 ± 1.02	0.65 ± 0.08a	1.64 ± 0.21a
T4	6.55 ± 0.06	11.48 ± 0.19b	76.12 ± 2.89b	1.35 ± 0.04b	87.92 ± 1.91ab	11.52 ± 0.65	0.43 ± 0.02b	1.12 ± 0.04b

*Values are means ± SD of three replicates. Values in the same column with different superscripts differ significantly (*n* = 3; *P* < 0.05). IBW, initial body weight; FBW, final body weight; WG, weight gain; SGR, specific growth ratio; FI, feed intake; FE, feed efficiency; PER, protein efficiency ratio.*

### Muscle Composition

The proximate composition of shrimp muscle in all treatments is shown in Table 5. No significant differences were found in moisture, crude ash, or lipid contents of muscle among all treatments (*P* > 0.05). Crude protein contents of shrimp muscle in T4 treatment were significantly lower than that of shrimp in the T1 treatment (*P* < 0.05) but without significant differences with shrimp in the T2 and T3 treatments (*P* > 0.05).

**TABLE 5:** Effects of different feeding strategies on muscle composition of *Litopenaeus vannamei.*

Parameters (%)	Feeding strategies			
	T1	T2	T3	T4
Moisture	73.04 ± 0.95	74.07 ± 0.70	73.67 ± 0.47	74.49 ± 0.58
Protein	23.25 ± 0.35a	22.39 ± 0.58ab	22.71 ± 0.39ab	21.92 ± 0.99b
Lipid	0.8 ± 0.02	0.79 ± 0.02	0.75 ± 0.02	0.77 ± 0.03
Ash	1.56 ± 0.09	1.48 ± 0.07	1.52 ± 0.04	1.59 ± 0.04

*Values are means ± SD of three replicates. Values in the same row with different superscripts differ significantly (*n* = 3; *P* < 0.05).*

### Digestive Enzyme Activity and Gene Expression Level

**FIGURE 1:**
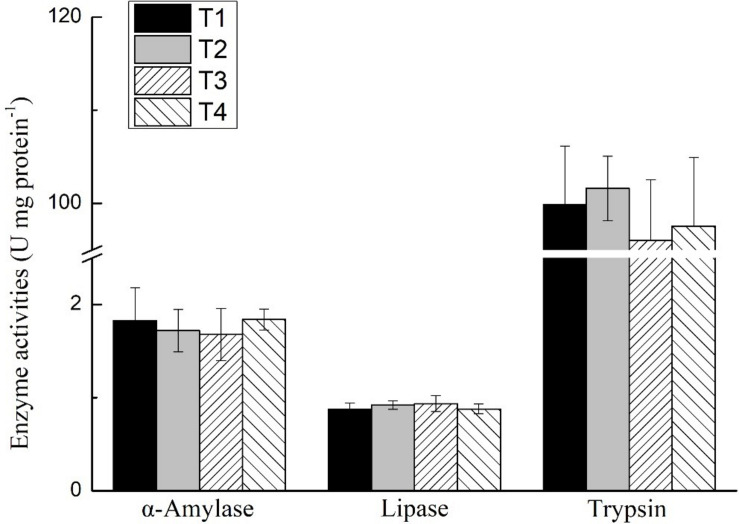
Effects of different feeding strategies on digestive enzyme activities of *Litopenaeus vannamei*. Values are means, with standard deviation represented by vertical bars.

**FIGURE 2:**
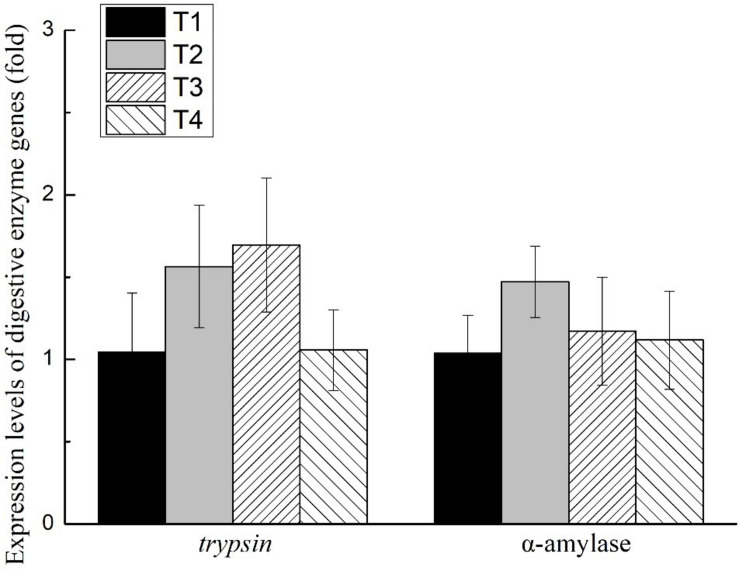
Effects of different feeding strategies on expression level of digestive enzyme genes in *Litopenaeus vannamei*. Values are means, with standard deviation represented by vertical bars.

The activities of trypsin, α-amylase, and lipase of the shrimp hepatopancreas in all treatments are shown in Figure 1. No significant differences were found in the activities of trypsin, α-amylase, or lipase of shrimp in any treatments (*P* > 0.05). The expression level of *trypsin* and α*-amylase* of the shrimp hepatopancreas in all treatments is shown in Figure 2. No significant differences were found in the expression of *trypsin* and α*-amylase* in any treatments (*P* > 0.05).

### Expression Levels of the mTOR Signaling Pathway Gene

**FIGURE 3:**
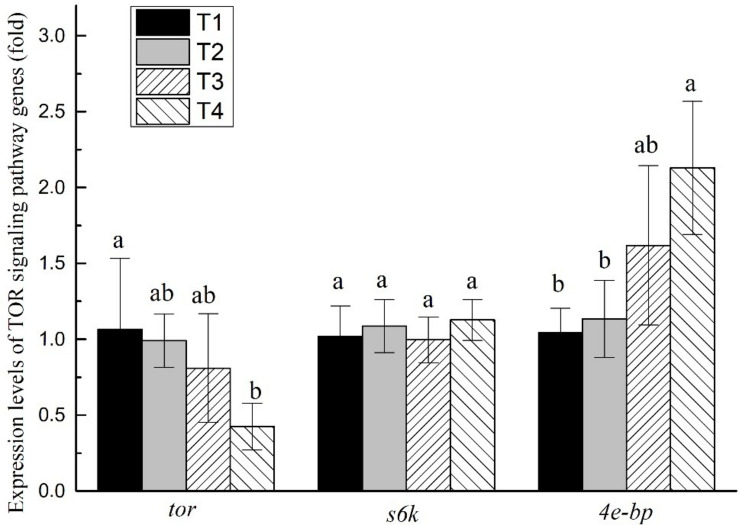
Effects of different feeding strategies on gene expression levels related to mTOR signaling pathway in *Litopenaeus vannamei*. Values are means, with standard deviation represented by vertical bars. Mean values with different letters are significantly different (*P* < 0.05).

The mTOR signaling pathway gene expression levels in all experiments were affected by different feeding strategies (*P* < 0.05; Figure 3). The *tor* expression level of shrimp muscle in T4 treatment was significantly lower than that of shrimp in the T1 treatment (*P* < 0.05) but without significant differences from shrimp in the T2 and T3 treatments (*P* > 0.05). The *4e-bp* expression levels of shrimp muscle in the T4 treatment were significantly higher than that of shrimp in the T1 and T2 treatments, but without significant differences from shrimp in the T3 treatment (*P* > 0.05). The expression level of *s6k* did not show significant differences in any of the treatments (*P* > 0.05).

## Discussion

The intensive high-density culture mode is widely used in shrimp culturing in China. From the perspective of practical application, a protein restriction cycle strategy under high-density culture conditions was conducted to evaluate the growth and digestive performance of *L. vannamei* in this study. The results indicated that the application of a suitable protein-restriction cycle strategy could not adversely affect the growth performance of *L. vannamei* in 42 days. The growth performance (FBW, GW, SGR), FE and PER of shrimp in the T2 and T3 treatments were not significantly different with that of shrimp in the T1 treatment (control group). Dong et al. (2013) demonstrated that moderate protein restriction and subsequent recovery can compensate completely in terms of final body weight and growth rate of *Pelteobagrus fulvidraco*. Similar results also have been reported in *Fenneropenaeus chinensis* (Wu and Dong, 2002), *Oncorhynchus mykiss* (Sevgili et al., 2012), and *Pelodiscus sinensis* (Xie et al., 2012). However, poor growth performance of FBW, GW, SGR, FE, and PER of shrimp was observed in the T4 treatment group. This result indicated that the compensatory growth response of *L. vannamei* is related to the duration of protein-restriction. Survival of shrimp in the T2–T4 treatments were higher than that of shrimp in the T1 treatment. The survival rate of the shrimp in the T1 treatment group is about 80%, which is similar to that of shrimp cultured under high density in practical conditions. The results indicated that short-term protein restriction can improve the survival rate of *L. vannamei* under high-density culture conditions. Pursuing high yield is the main aim of aquaculture, so the protein level of a commercial diet is at a relatively high level in order to promote the growth of aquatic animals. However, sustained high protein intake may impose a physiological burden on the hepatopancreas and intestine of *L. vannamei*, thus affecting immunity and survival rate.

In this study, there were no significant differences in the lipid, moisture, and ash contents in the muscle of shrimp in any treatment, but protein content decreased significantly in T4 treatment. Similar results have been reported by Wu and Dong (2002). Their research indicated that 15% protein level restriction and subsequent recovery significantly reduced the protein content of *F. chinensis*, while 30% protein restriction had no significant effect on protein content. However, in an experiment on *P. fulvidraco* (initial body weight of 8.33 ± 0.01 g), there were no significant differences in the whole-body protein content between protein-restricted treatment (320 g kg^–1^: weeks 1–4; 390 g kg^–1^: weeks 5–8) and control treatment (390 g kg^–1^: weeks 1–8) (Dong et al., 2013). These different results between our present study and Dong et al. (2013) may be attributed to the severity and duration of protein restriction, experimental conditions (culture density and species size), and interspecific differences. In view of the results of growth performance, the T4 treatment group’s diet protein is not enough to supply growth performance, so no excess protein was deposited in the muscle.

The activity of digestive enzymes plays a key role in the digestion and absorption of nutrients, animal growth and the adaptability to the environment (Shao et al., 2018). Trypsin, lipase, and α-amylase are considered to be the main digestive enzymes of shrimp that can affect the digestion and absorption of food (Muhlia-Almazán et al., 2013). In this study, no significant differences were found in the activities and gene expression level of trypsin, α-amylase, or lipase of shrimp in any treatments. Generally, the increase of digestive enzyme activity can contribute to the digestion and absorption of nutrients in aquatic animals, thus promoting growth performance (Aguila et al., 2007). However, in this study, the activity of digestive enzymes did not vary with different feeding strategies. We speculate that samples were collected a week after protein recovery, so digestive enzyme activity may have returned to a normal level during protein recovery. Undoubtedly, more studies are needed to address this issue. In the next study, we will collect samples after protein restriction and protein recovery to further explore the role of digestive enzymes in the protein restriction strategy for shrimp growth performance.

The mTOR signaling pathway plays a key role in cell survival, growth, proliferation, apoptosis, and protein synthesis and degradation (Wullschleger et al., 2006; Zhao et al., 2017). It can be activated by a variety of stimuli, such as growth factors, nutrients, energy, and stress signals (Pópulo et al., 2012). The core genes of the mTOR signaling pathway are *tor*, while *s6k* and *4e-bp* are two key downstream genes. mTOR signaling pathway is positively regulated by *tor* and *s6k*, and inversely regulated by *4e-bp* (Dazert and Hall, 2011; Laplante and Sabatini, 2012). In this study, the expression level of *tor* in T4 treatment was lower than in other treatments, while *4e-bp* was higher than in any other treatment. The results indicated that the mTOR signaling pathway was inhibited in the T4 treatment. Growth performance and muscle protein content were also significantly lower in the T4 treatment than in other treatments. Therefore, we speculate that long-term protein restriction may inhibit the mTOR signaling pathway, reduce muscle protein synthesis, and shrimp growth performance. The results also showed that the growth performance and PER in the T4 treatment were significantly lower than in any other treatment and may not be affected by digestive enzyme activity but by the regulation of mTOR signaling pathway.

## Conclusion

*L. vannamei* under high-density culture conditions were fed diets of 36% protein for 1 week and then 43% protein for 1 week (T3 treatment) or 2 weeks (T2 treatment), and no adverse effects on growth performance (FBW, GW, SGR), FE, PER, muscle composition, digestive enzyme activity, or the expression level of the mTOR signaling pathway related genes were observed. However, when shrimp were fed 36% protein diets for 2 weeks and subsequent recovery (T4 treatment), poor growth performance (FBW, GW, SGR), FE, PER, and muscle protein content were obtained. Moreover, the mTOR signaling pathway was inhibited in the T4 treatment. One week of protein restriction could induce the compensatory growth response of *L. vannamei*, while two weeks of protein restriction significantly inhibited growth performance. In order to save on protein and feed costs, giving shrimp a protein-restricted diet for 1 week with subsequent refeeding (T2 and T3) is suitable for shrimp under high-density conditions.

## Data Availability Statement

The original contributions presented in the study are included in the article/supplementary material, further inquiries can be directed to the corresponding author/s.

## Ethics Statement

The animal study was reviewed and approved by the Experimental Animal Ethics Committee of Fujian Agriculture and Forestry University.

## Author Contributions

JS and WZha made most contributions to this research, such as experiment design, sample collection, analysis of data, drafting the manuscript, etc. HL made contributions to data analysis and drafting the manuscript. WZhu participated in sample collection and analysis. XY contributed to sample collection and data analysis. All authors contributed to the article and approved the submitted version.

## Conflict of Interest

The authors declare that the research was conducted in the absence of any commercial or financial relationships that could be construed as a potential conflict of interest.
